# How can we measure resource quality when resources differ in many ways? Deconstructing shelter quality in a social fish

**DOI:** 10.1002/ece3.70146

**Published:** 2024-08-12

**Authors:** Aneesh P. H. Bose, Tomas Brodin, Cyprian Katongo, Lwabanya Mabo, Alex Jordan

**Affiliations:** ^1^ Department of Wildlife, Fish & Environmental Studies Swedish University of Agricultural Sciences (SLU) Umeå Sweden; ^2^ Behavioural Evolution Research Group Max Planck Institute of Animal Behavior Konstanz Germany; ^3^ Centre for the Advanced Study of Collective Behaviour University of Konstanz Konstanz Germany; ^4^ Department of Biological Sciences University of Zambia Lusaka Zambia; ^5^ Lake Tanganyika Research Unit, Department of Fisheries Ministry of Fisheries and Livestock Mpulungu Zambia; ^6^ Department of Biology University of Konstanz Konstanz Germany

**Keywords:** niche partitioning, resource competition, shelter choice, shell dwelling, shelter architecture, social status

## Abstract

Resource quality is an important concept in ecology and evolution that attempts to capture the fitness benefits a resource affords to an organism. Yet “quality” is a multivariate concept, potentially affected by many variables pertaining to the resource, its surroundings, and the resource chooser. Researchers often use a small number of proxy variables to simplify their estimation of resource quality, but without vetting their proxies against a wider set of potential quality estimators this approach risks overlooking potentially important characteristics that can explain patterns of resource use in their study systems. Here we used *Neolamprologus multifasciatus*, a group‐living cichlid fish that utilizes empty snail shells as shelter resources, to examine how shells were used by, and partitioned among, group members in relation to a range of attributes, including shell size, intactness, texture, spatial position, and usage by heterospecifics. This approach generated a comprehensive picture of what characteristics contribute to the attractiveness and quality of each shell resource, confirming the importance of two previously proposed shell characteristics, size and intactness, but highlighting the influences of other unexplored variables, including shell spatial position and usage by heterospecifics. We also present a generally applicable “resource attractiveness index” as a means to estimate resource quality based on resource choice data. This index incorporates information from any number of resource characteristics and is of particular use when researchers wish to quantify resource value, but many characteristics jointly contribute to the value and attractiveness of the resource.

## Graphical abstract

Resource quality is an important but difficult‐to‐quantify concept in ecology and evolution. Here we generate a comprehensive picture of the characteristics that contribute to the attractiveness and quality of shelter resources used by *Neolamprologus multifasciatus*, a group‐living cichlid fish. We highlight how multiple resource traits can jointly determine the quality and attractiveness of a resource, and we provide a generally applicable approach to quantifying resource quality that can be used in many study systems.

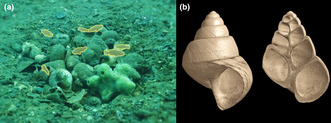

## 1 INTRODUCTION

Resource quality is a concept that attempts to capture the fitness yields associated with accessing a particular resource, and its corresponding prediction is that high‐quality resources are more attractive than low‐quality resources. Yet, “quality” is a multivariate concept; the quality of a resource can be influenced by potentially many different variables that can interact in complex ways (Bertness, 1981; Ens et al., 1992; Franks et al., 2003). Resource quality can therefore be challenging to quantify directly, and so proxies for quality are often relied upon. Empirical studies frequently reduce resource quality down to one or two variables that are presumed to be consequential for individual fitness (Kelly, 2008). For example, estimates of insect abundance were used as proxies for territory quality in Seychelles warblers, *Acrocephalus sechellensis* (van de Crommenacker et al., 2011). In threespine stickleback, *Gasterosteus aculeatus*, male territory quality has been estimated by its area and vegetation cover (Candolin & Voigt, 2001). Koenig et al. (1998) measured food quality for leaf‐eating primates in terms of the concentration of extractable proteins and sugars in edible leaves. Nest quality has been quantified using proxies for insulation capacity in birds (e.g., penduline tits, *Remiz pendulinus*, Hoi et al., 1994) and the space available for egg‐laying in fishes (e.g., sand goby, *Pomatoschistus minutus*, Lehtonen et al., 2007; *Pundamilia* spp., Dijkstra et al., 2008; plainfin midshipman fish, *Porichthys notatus*, Bose et al., 2018). Yet, by reducing resource quality down to only a small number of proxy variables, researchers risk overlooking characteristics of resources that could be integral to explaining the behavior of their study species. We are therefore lacking studies that consider resource quality from a more multivariate perspective, testing which characteristics contribute most to resource value and attractiveness. Ultimately, this will help clarify which variables are most useful for adequately capturing resource quality.

Accurately estimating resource quality is important because resources are often non‐randomly partitioned among conspecific and heterospecific individuals under natural conditions, generating a myriad of ecological and evolutionary consequences that drive natural and sexual selection. Partitioning can occur either as a result of competition or individuals differing in their requirements, motivations, or preferences. Competitively superior individuals are expected to outcompete inferior or less motivated individuals to obtain the highest quality resources. Within animal social groups, dominant individuals frequently receive priority access to resources at the expense of subordinates (e.g., Hanuman langurs, *Presbytis entellus*, Koenig et al., 1998; lions, *Panthera leo*, Packer et al., 2001; rhesus macaques, *Macaca mulatta*, Rebout et al., 2017). Individuals in a group can also differ in their preferences for resource types, which may be a direct consequence of interactions with group mates. For example, in cooperatively breeding scrub jays, *Aphelocoma coerulescens*, individual preferences for food‐caching sites differ with age, experience, and social rank as a way to avoid pilfering from conspecifics (Fuirst et al., 2020). In gregarious cockroaches, shelter preferences change with ontogeny, but can also be affected by social context (Jeanson & Deneubourg, 2007). Thus, it is not uncommon to observe group‐members that vary in sex, age, body size, or social status use resources that diverge in quantity or quality, though the challenge still remains as how to accurately quantify resource quality.

Here, we examine the degree to which a single critical resource type varies in quality, and how it is partitioned among social group members that differ in age, sex, and social status in a cichlid fish, *Neolamprologus multifasciatus*. Using this highly tractable system, we can take multiple measurements of their essential resources, empty snail shells, and record the usage of these shells by all social group members. Groups of this species consist of a single dominant male as well as several females, subordinate males, and juveniles and can range up to ~20 fish (Bose, Dabernig‐Heinz, et al., 2022a; Bose, Koch, et al., 2022b; Kohler, 1998). *Neolamprologus multifasciatus* is endemic to Lake Tanganyika, East Africa, and live in habitats called “shell beds,” which are characterized by massive accumulations of snail shells. Each group controls a territory on the lake floor containing a collection of empty *Neothauma tanganyicense* snail shells that they excavate from the sediment. Each fish (unless they are still receiving parental care) will occupy a single shell from its group's limited supply, which we term its “home shell” (Gübel et al., 2021). These shells serve as shelters and are vital to the survival and reproduction of all group members, thus all individuals are expected to be motivated to choose a high quality shell as their own. Unlike larger shell‐breeding cichlid species like *Lamprologus callipterus* (Maan & Taborsky, 2008; Schütz & Taborsky, 2005), *N. multifasciatus* are unable to physically transport shells, and so they are constrained to select from the pool of shells on their territory. Though the shells are highly consistent in shape, they vary in size and intactness (McGlue et al., 2010), and *N. multifasciatus* strongly prefer to reside in larger and more intact shells (Bose et al., 2020). However, *N. tanganyicense* shells also vary with respect to other characteristics that could conceivably influence their quality as a shelter. We systematically examined these characteristics to uncover a more comprehensive picture of what contributes to resource quality in this system. Because *N. multifasciatus* group members vary in competitive ability (and perhaps also resource preference), we predicted that shells would be partitioned between the sexes, age classes, and social statuses of the fish. We predicted that the competitiveness of shell occupants would co‐vary with the quality of the shell they were residing in, analogous to an ideal despotic distribution (Fretwell, 1972), with dominant males occupying the highest quality shells available in the group, followed by subordinate males, then females, and finally juveniles (predictions are based on the average body size for each type of group member, Bose, Dabernig‐Heinz, et al., 2022a; Bose, Koch, et al., 2022b; present study). We also computed a “resource attractiveness index,” which rates each resource according to how attractive it is to choosers, and does so by integrating information about all the various features measured from each resource.

## 2 METHODS

During September–October 2021 and April–May 2023, we haphazardly selected 41 *N. multifasciatus* social groups (30 groups in 2021 and 11 groups in 2023) while on SCUBA from a dense colony located near Mutondwe Island, Lake Tanganyika (8°42′48.8″ S, 31°07′23.3″ E, 10–17 m deep). Upon selection of a social group, we systematically collected and measured all *N. tanganyicense* shells within the territory, including the residents of those shells. *N. multifasciatus* social groups control territories that always contain at least at many shells as they have group members (Kohler, 1998). Though, some shells within *N. multifasciatus* territories are unusable as shelters because: (a) the shell belonged to a living *N. tanganyicense* snail, (b) the shell was filled completely with sediment, (c) the shell entrance was blocked by other shells or shell fragments, or (d) the shell itself is too broken. When the fish excavate shells from the sand they create pit‐shaped territories filled with layers of shells. Shells within a territory can therefore be categorized as either surface shells, at the top and in‐line with the lake floor, or basement shells, which are underneath and can be partially buried. As we were collecting, we recorded whether each shell was located at the surface or basement level of each territory.

Since the fish retreat into their respective “home shell” when approached by a SCUBA diver (Gübel et al., 2021), when we collected the shells, we simultaneously collected their residents. We transported the shells, and any residents inside, to the surface for more detailed measurements. One scorer estimated the intactness of every shell by visual inspection. This is because shells can accumulate holes and fractures over time due to weathering (Bose et al., 2020; McGlue et al., 2010). We expected intact shells to be more sought after, as previously shown in laboratory preference trials (Bose et al., 2020), because intact shells offer more structural integrity and likely more protection from predators than less intact shells, as has been shown for hermit crabs (*Pagurus pollicaris*, McClintock, 1985; *Pagurus longicarpus*, Rotjan et al., 2004). More intact shells may also provide additional safety from predators like *N. tetracanthus*, which can extract fish (and shrimp) from shells by creating a suction seal between their mouths and the shell aperture (AB personal observations). The scorer assigned each shell an intactness rating ranging from 30% to 100% (in 10% intervals) corresponding to the percent of the shell wall that was still intact (note that shells with low intactness ratings were simply missing portions of their outer wall and could still be picked up). Shells judged to be less than 30% intact were deemed to be too broken to be usable as shelters. Laboratory preference trials have revealed that large shells may be more sought after than small shells (Bose et al., 2020, likely because they provide extra space and allow fish to reside deeper in the shell away from the entrance where predators can access), and because of this one scorer also measured shell size. Specifically, we measured the diameter of the major axis of each shell's entrance (using a ruler, to the nearest 0.1 cm) as a proxy for shell size. This was done because some shells were not intact enough to measure their full length, yet entrance size could still be measured from all shells that were at least 30% intact. Entrance size grows isometrically and in close correlation with shell size (Bose et al., 2020), making it a reliable proxy variable. One scorer also visually assessed whether each shell had smooth or rough walls. *Neothauma tanganyicense* shells in Lake Tanganyika vary enormously in age, and such age variability is clearly visible as many, presumably older, shells have undergone noticeable stromatolitic encrustation, bearing thick, rough, and cemented walls, while other shells still possess their original thinner, smoother walls (see McGlue et al., 2010). Approximately 7.2% of the shells we collected had smooth walls. We expected shell smoothness to influence shelter value as smooth walls may permit fish to move further into the shell to avoid predators or to better adhere their eggs to the inside of the shell. Next, we recorded whether a shell was covered by an encrusting sponge. Sponges are abundant in certain regions of the shell bed where they can encrust large surfaces, including entire shells (McGlue et al., 2010), sometimes affixing them to nearby substrata (e.g., rocks or other shells). We expected sponge cover to affect resource value if by fixing the shell in place, the sponges make the shells more difficult to move or enter by predators or shell competitors (e.g., *Mastacembelus* spp., *Neolamprologus callipterus*, etc.). Approximately 4.6% of the shells we collected had sponge cover.

Each shell was carefully broken open with a small mallet to record whether it was occupied. Resident *N. multifasciatus* were sexed by inspection of their urogenital papillae, measured for standard length (SL) using a ruler (to the nearest 0.1 cm), and recorded as either adults or juveniles based on the presence of distinct banding along the sides of the body denoting sexual maturity (Bose, Dabernig‐Heinz, et al., 2022a; Kohler, 1998). Adult males were also categorized as either dominant or subordinate, with the largest male in each group always being dominant (Bose et al. 2021, Bose, Dabernig‐Heinz, et al., 2022a, Bose, Koch, et al., 2022b, Bose et al., 2023; Jordan et al., 2016; Kohler, 1998). While dominant males freely traverse their group's whole territory space, other group members tend to remain in their own separate sub‐territories (Bose et al. 2021, 2023). Shell occupants that were not *N. multifasciatus*, were either a heterospecific fish, which we identified down to species, a shrimp or a crab. Shrimp were measured for total length (TL), and crabs were measured across the widest portion of their carapace. Note that crabs and shrimp were of comparable body size to *N. multifasciatus* (see Results), and so it is unlikely that the fish feed on these crustaceans, but rather coexist or compete with them for space within the shells. In 2021, all shell occupants were released back to the shell bed the following day by a SCUBA diver. The fish were returned to the location of their capture and released in the vicinity of empty, available shells, which the fish would colonize. In 2023, the shell occupants were used in subsequent behavioral experiments in the laboratory.

In total, we collected 1256 shells (that were at least 30% intact) from the 41 social groups. Of these shells, 57 belonged to living *N. tanganyicense* snails, 138 were either filled with sediment or had their entrances otherwise blocked, and four were occupied by heterospecific fish (a juvenile *Altolamprologus compressiceps*, a juvenile *Phyllonemus filinemus*, and two juvenile *Telmatochromis temporalis*). The four shells containing heterospecific fish were not shared with any *N. multifasciatus*, and so we assumed that if a shell was occupied by a heterospecific fish, it would not be available for occupancy by *N. multifasciatus*. Thus, 1057 shells were ultimately deemed potentially useable as shelter resources, and were examined further in our analyses.

All methods adhered to the ASAB/ABS Guidelines for the Use of Animals in Research. Fieldwork was carried out with approval from the Fisheries Department at the Ministry of Fisheries and Livestock Zambia, under a study permit issued by the government of Zambia (No. SP260718/7‐21) and in conjunction with a memorandum of understanding with the University of Zambia (MOU 101/14/11). *N. multifasciatus* is listed as “Least Concern” on the IUCN Red List of Threatened Species. When returning the fish to the wild, the fish were released near to where they were captured and in areas with plenty of available shells, which the fish would quickly swim toward and settle into. No predation events were observed after releasing the fish.

### 2.1 Statistical analyses

We tested how shells that were occupied by *N. multifasciatus* individuals differed from those that were unoccupied. We did this by fitting a generalized linear mixed effects model (GLMM, which we term the “shell occupancy model,” see below) assuming a binomial family and a logit link function (using the R package “glmmTMB,” Brooks et al., 2017). We fitted a binary response variable indicating whether a shell was occupied by a *N. multifasciatus* (1 = Occupied, 0 = Unoccupied). For predictor variables, we included “shell location” (categorical, surface vs. basement), “shell intactness” (continuous, scaled to have a mean of 0 and standard deviation of 1), “shell entrance size” (continuous, also scaled to have a mean of 0 and standard deviation of 1), “shrimp presence” (binary), “crab presence” (binary), “encrusting sponge presence” (binary), and “shell smoothness” (categorical, rough vs. smooth). Note that shell intactness and shell entrance size were scaled (mean‐centered and standardized by dividing by their standard deviation) so that we could compare their parameter estimates and 95% confidence intervals for overlap to assess their relative importance in contributing to shell occupancy (Schielzeth, 2010). Two‐way interactions between shell location, intactness, and entrance size were included in the model but were dropped from the final model if they did not improve model fit. We used a likelihood ratio test to determine if an interaction improved model fit. We included a random intercept of “group ID” to account for non‐independence arising from shared group membership (i.e., each group can only choose from the set of shells they have available to them on their territory). Model diagnostics were checked using the R package “DHARMa” (Hartig, 2022) and multicollinearity was checked by calculating variance inflation factors using the R package “performance” (Lüdecke et al., 2021).

Next, we tested whether shells at the surface of a territory displayed different characteristics than the shells in the basement levels. To do this, we fitted a GLMM assuming a binomial family and a logit link function and included shell location as the response variable. We then included “shell intactness” (scaled), “shell entrance size” (scaled), “shrimp presence,” “crab presence,” “encrusting sponge presence,” and “shell smoothness” as predictor variables, and “group ID” as a random intercept. We tested for the two‐way interaction between entrance size and intactness, but dropped it from the final model because it did not significantly improve model fit as assessed with a likelihood ratio test.

We then fitted a GLMM to ask whether dominant males, subordinate males, females, or juveniles were more likely than others to live in the basement or surface. We assumed a binomial family and a logit link function and included “shell location” as a binary response variable. We included ‘group member type’ as a categorical predictor variable (four levels: dominant male, subordinate male, female, juvenile), and “group ID” as a random intercept. We then used pairwise contrasts and univariate *p*‐values to compare each group member type to one another (using the “glht” function in the Rpackage “multcomp,” Hothorn et al., 2008).

Next, we compared shell characteristics between those shells occupied by dominant males, subordinate males, females, and juveniles. We fitted a multinomial baseline‐category logit model (using the “mblogit” function in the R package “mclogit,” Elff, 2021). This model examines how different predictors affect the odds of falling into one response category relative to a baseline category. We set the response variable to be “group member type” (dominant male, subordinate male, female, juvenile), and releveled the baseline category to capture all pairwise comparisons. As predictor variables, we included “shell location,” “shell intactness” (scaled), “shell entrance size” (scaled), “shrimp presence” (binary), “shell smoothness” (categorical, rough vs. smooth), and “group ID” (categorical). Note that because some configurations of the response and predictor variables were very rare or did not occur, this model had to be simplified to reach convergence. For example, of 26 shells that contained crabs, only three were shared with *N. multifasciatus* (one female, two juveniles). We therefore removed crab presence, and also encrusting sponge presence, from the model. The interaction term between shell intactness and shell location, which was found to significantly increase model fit in the above GLMM, did not do so here based on an analysis of deviance, and was therefore also not included. We checked for multicollinearity by calculating generalized variance inflation factors.

### 2.2 Resource attractiveness index

The above analyses examined the roles of different shell characteristics in explaining shell occupancy by *N. multifasciatus*, providing a glimpse into which features contribute to shell attractiveness and value in this system. We found that more than one feature influenced resource choice in our study, and because of this, we chose to calculate a single composite measure of resource attractiveness that incorporates these multiple features. We therefore calculated a “resource attractiveness index” for each shell in our dataset, and we explain how such an index can be generalized to any study system involving choice between complex resources.

Our approach follows the philosophy laid out by Johnson (1980), which argues that understanding resource usage (or non‐usage) requires accounting for all alternatives that are available to a chooser and the ways in which investigators deem the alternatives to differ from one another. We began by extracting predictions from the “shell occupancy model” to yield the probability that each shell, with its given set of characteristics, would be occupied by a *N. multifasciatus* (using the “predict” function in R). This step produced probabilities that ranged from ~0.002 to ~0.626 across the shells in our dataset, generally capturing shells with poor characteristics at one end (e.g., small, broken shells) and shells with desirable characteristics at the other end (e.g., large, intact shells). We then ran this model prediction step again, but we randomized which shells were occupied (randomizations occurred within groups to control for differences among groups in the quality of shells they had available to them). We repeated this randomization procedure 1000 times and averaged the resulting models' occupancy predictions for each shell separately. The average of the randomized predicted occupancy probabilities for each shell was then subtracted from that shell's observed probability of occupancy. This step removes variation in the predicted probabilities of shell occupancy that could be attributable to random choice. Thus, the resource attractiveness index gives a value above (positive) or below (negative) random chance that any given resource will be chosen. The final step was to scale this variable according to each social group's set of shells in their territory. We did this by mean‐centering the resource attractiveness score of each shell and dividing by the standard deviation for each group separately in our dataset.

Finally, we repeated the previous multinomial baseline‐category logit model, but we replaced all the previous shell characteristic variables (i.e., shell location, intactness, entrance size, shrimp presence, and shell smoothness) with our shell attractiveness index (continuous).

## 3 RESULTS

**FIGURE 1:**
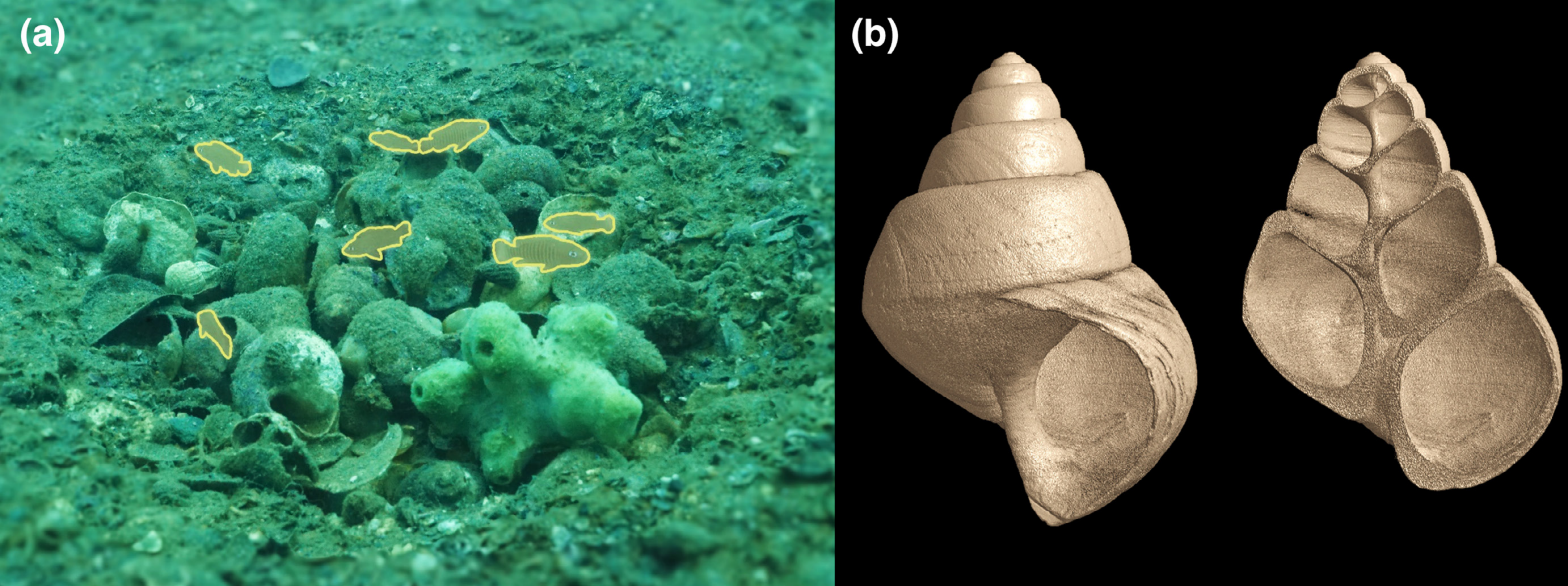
(a) Photograph depicts a *N. multifasciatus* group in the wild (individuals highlighted). The fish excavate shells from the sand, creating depressions on the lake floor, which contain their collections of unearthed, empty shells (photo credit: Aneesh Bose). (b) Micro‐CT scan of *N. tanganyicense* snail shell used as shelters by *N. multifasciatus* (photo credit: Fabrizia Ronco).

The 41 *N. multifasciatus* social groups each contained one dominant male (average standard length ± std. dev. = 2.73 ± 0.16 cm), zero to two subordinate males (2.38 ± 0.14 cm), zero to seven females (1.92 ± 0.10 cm), and zero to nine juveniles (1.64 ± 0.28 cm). Juveniles are immature individuals measuring at least 1 cm in standard length, which is when they reach independence from maternal care and occupy shells of their own (Bose, Dabernig‐Heinz, et al., 2022a; Bose, Koch, et al., 2022b). In total, we documented 251 *N. multifasciatus* males, females, and juveniles occupying 226 shells (Figure 1). Twelve of these individuals evaded our capture during sampling so their home shells unfortunately could not be recorded (one dominant male, one subordinate male, two females, and eight juveniles). Adults were never found sharing a shell together, but we observed several cases of adults sharing a shell with juveniles or multiple juveniles sharing a shell together (three dominant males each shared their shell with a juvenile, one subordinate male shared its shell with a juvenile, five females each shared their shells with one to two juveniles, and three shells contained two juveniles).

Each social group had 25.8 ± 15.8 useable shells (average ± std. dev., range = 4–76 shells). Average (± std. dev.) entrance size was 2.00 ± 0.22 cm (range = 1.1–2.7 cm), and intactness was rated as 76.7 ± 23.0% (range = 30%–100%, median = 80%). Shell entrance size was not correlated with shell intactness (Pearson correlation coefficient = −0.048). Thirty‐five shells were occupied by crabs (average carapace width ± std. dev. = 0.81 ± 0.28 cm), and in three of these cases a *N. multifasciatus* shared a shell with a crab (one female, two juveniles). Forty‐three shells contained shrimp (length = 1.79 ± 0.32 cm), and in nine of these cases the shrimp were cohabiting a shell with a *N. multifasciatus* (two dominant males, one subordinate male, two females, four juveniles). Forty‐nine shells were covered in encrusting sponges, of which four were occupied by *N. multifasciatus* individuals (two dominant males, two juveniles). Seventy‐six shells had smooth walls, and 24 of them were occupied by *N. multifasciatus* (three dominant males, four subordinate males, 10 females, seven juveniles). Finally, 282 shells were located in the basements of the territories, relative to 775 located at the surface level.

**FIGURE 2:**
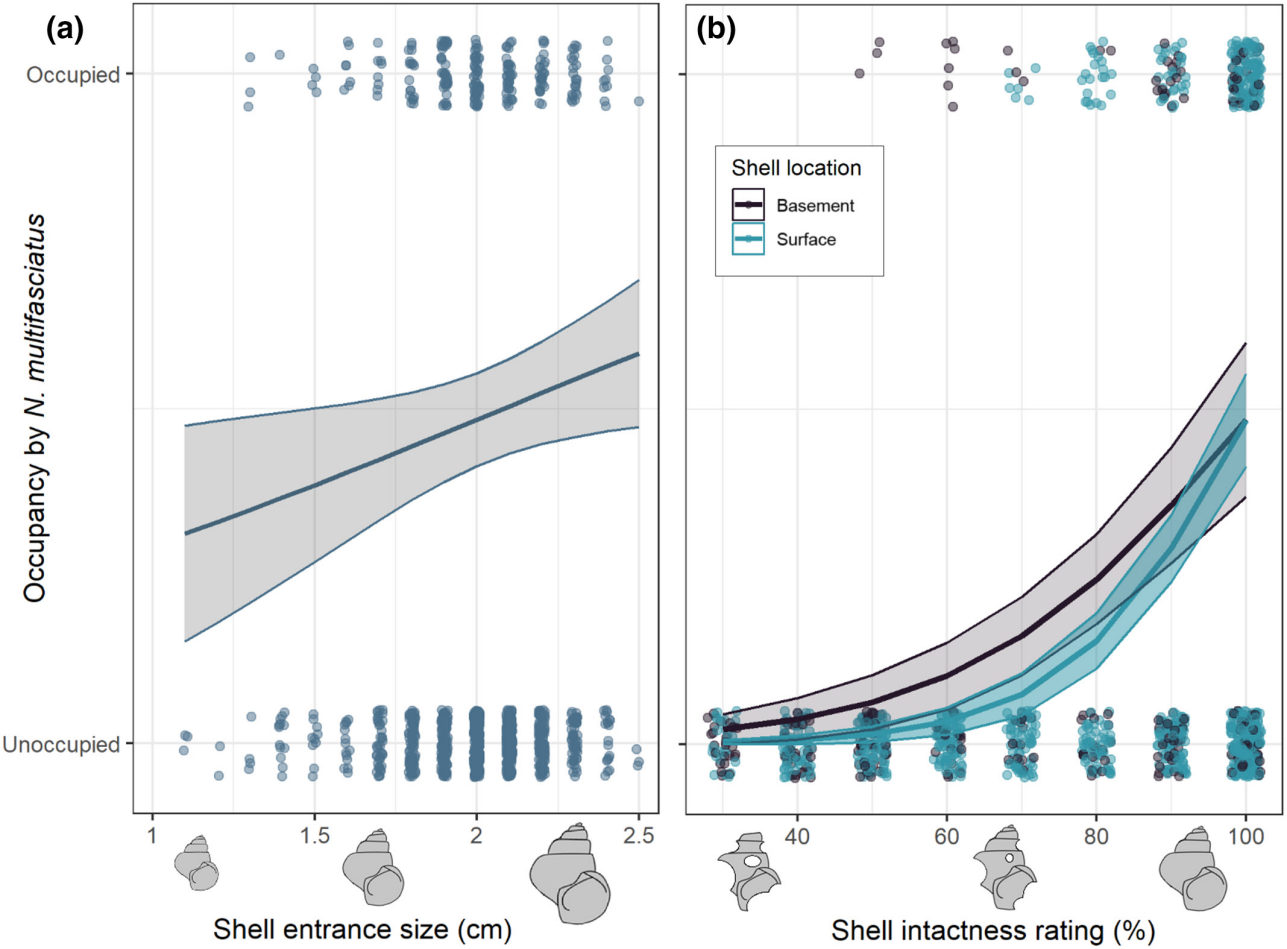
(a) Model predictions for the effects of shell size (measured using entrance size) on shell occupancy, overlaid on top of observed data (jittered slightly to improve visibility). (b) Model predictions for shell intactness at both surface and basement levels, overlaid on top of observed data (jittered). The plots show predicted model fits and 95% CIs. Shell drawings are given along x‐axes to visually depict size and intactness variation.

Larger shells were more likely to be occupied by *N. multifasciatus* group members (GLMM, entrance size: Est. ± SE = 0.17 ± 0.08, *z* = 2.08, *p* = .037, Figure 2a). We detected a significant interaction between shell intactness and location; shell intactness was positively associated with shell occupancy in the basement level (shell intactness: Est. ± SE = 1.21 ± 0.21, *z* = 5.90, *p* < .0001), and the log odds of occupancy increased more steeply with intactness at the surface level (interaction term: Est. ± SE = 0.66 ± 0.28, *z* = 2.37, *p* = .018, Figure 2b). The presence of either shrimp or crabs in a shell was negatively associated with *N. multifasciatus* occupancy (Est. ± SE = −0.93 ± 0.41, *z* = −2.25 *p* = .025; Est. ± SE = −1.94 ± 0.63, *z* = −3.09 *p* = .0020, respectively). Shell occupancy was not significantly related to the presence of encrusting sponges on the shell (Est. ± SE = −0.98 ± 0.56, *z* = −1.73, *p* = .084), nor with the smoothness of the shell walls (Est. ± SE = −0.43 ± 0.29, *z* = −1.50, *p* = .13).

**FIGURE 3:**
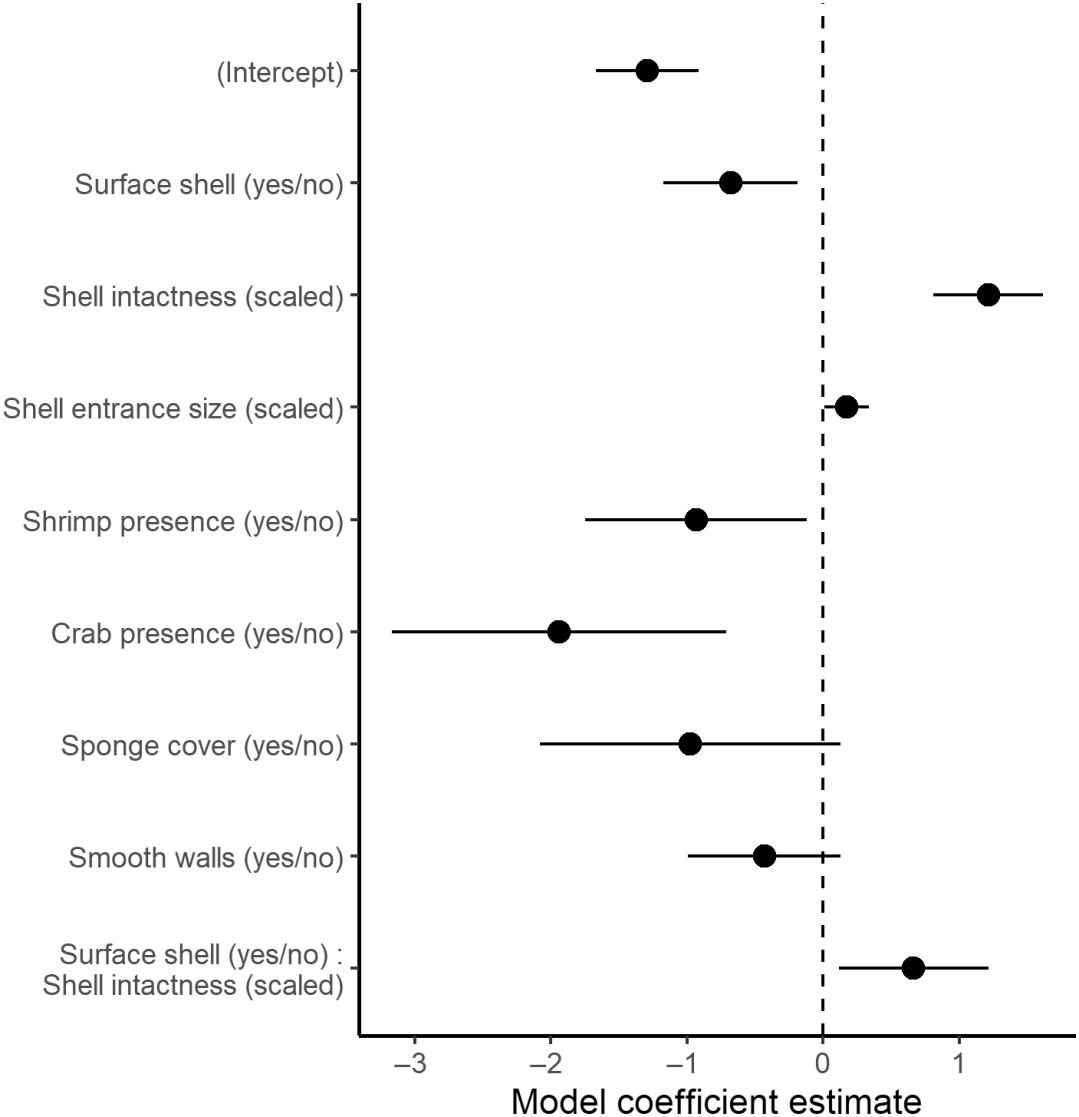
Coefficient plot depicting model estimates (dots) and 95% confidence intervals (solid bars) for the effect sizes of shell resource characteristics on shell occupancy by *N. multifasciatus*.

Overall, shell intactness was more important in predicting shell occupancy than shell entrance size. The effect size estimates and 95% confidence intervals for shell intactness (scaled variable, Est. = 1.21, 95% CI = 0.81–1.62) were higher and did not overlap with those for shell entrance size (scaled variable, Est. = 0.17, 95% CI = 0.010–0.34). Note that since our model includes an interaction term, the effect size for shell intactness is calculated at the basement level, which makes this comparison more conservative (Figure 3).

Compared to basement shells, surface shells were more intact (Est. ± SE = 0.48 ± 0.079, *z* = 6.09, *p* < .0001), larger (Est. ± SE = 0.44 ± 0.075, *z* = 5.88, *p* < .0001), and more likely to be covered in encrusting sponges (Est. ± SE = 1.29 ± 0.54, *z* = 2.39, *p* = .017). Surface shells were also less likely to have smooth walls than basement shells (Est. ± SE = −0.63 ± 0.30, *z* = −2.12, *p* = .034), and less likely to contain crabs (Est. ± SE = −0.97 ± 0.40, *z* = −2.42, *p* = .016). Surface shells did not differ from basement shells in their likelihood of housing shrimp (Est. ± SE = 0.045 ± 0.43, *z* = 0.11, *p* = .92).

Juveniles were more likely than dominant males (Est. ± SE = 1.60 ± 0.57, *z* = 2.81, *p* = .005) and subordinate males (Est. ± SE = 1.30 ± 0.66, *z* = 1.98, *p* = .048) to live in basement shells. Juveniles were not more likely than females to live in basement shells, though this difference approached significance (Est. ± SE = 0.70 ± 0.36, *z* = 1.93, *p* = .053). Dominant males, subordinate males, and females did not differ from one another in their probabilities of living in the basement (all |*z*| < 1.49, *p* > .13).

**FIGURE 4:**
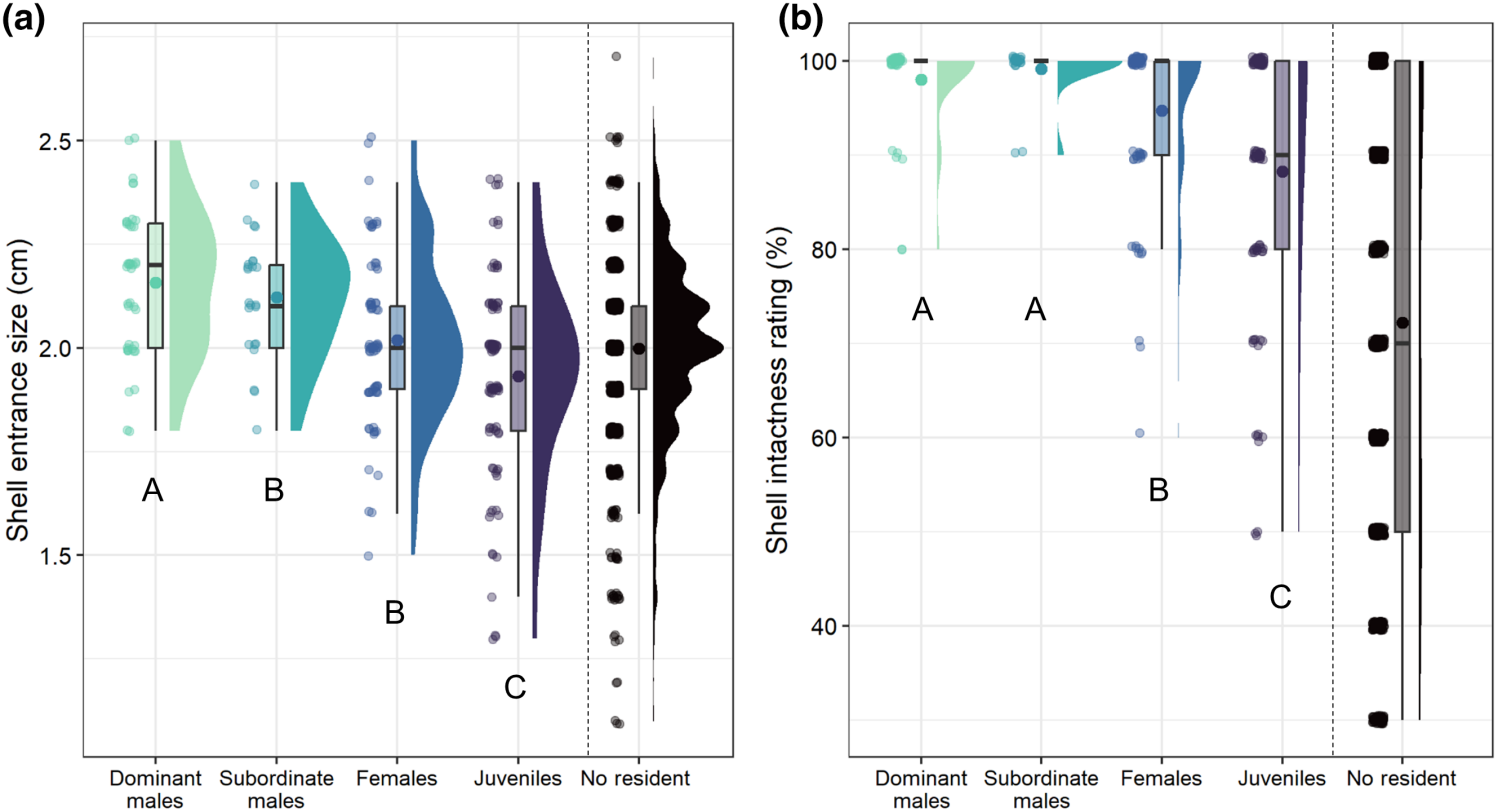
(a) Sizes of the shells occupied by different *N. multifasciatus* group members (shell entrance size was used as a proxy for overall size). Unoccupied shells are also shown to the right of the vertical dashed line, but were not included in the multinomial baseline‐category logit model. Data are visualized as points on the left (jittered slightly to improve visibility), box plots in the middle, and density plots on the right. Boxplots show medians (horizontal bar), means (large dot), the first and third quartiles (box), and the range of data within 1.5 interquartile distances above and below the interquartile (whiskers). Significant differences between group member types are given by different capital letters below each distribution. (b) Intactness ratings of the shells occupied by different *N. multifasciatus* group members.

The multinomial model revealed that as shell entrance size increased, shells were more likely to be occupied by dominant males than by subordinate males, females, or juveniles. As shell entrance size increased, shells were also more likely to be occupied by subordinate males and females than by juveniles (Figure 4a; Table 1). Increasing shell intactness did not significantly affect occupancy probabilities of dominant males relative to subordinate males, but did increase the probability of occupancy by males relative to females and juveniles (Figure 4b; Table 1).

**TABLE 1:** Output from a multinomial baseline‐category logit model, comparing characteristics of shells occupied by dominant males, subordinate males, females, and juveniles in *N. multifasciatus* social groups (the baseline category is given). Note that “GroupID” was included in the model as a fixed effect, but its output is not shown here.

Term	Estimate ± SE	*z*‐Value	*p*
Dominant male (baseline) versus subordinate male			
Intercept	−0.35 ± 2.48	−0.14	.89
Surface versus basement shell	0.20 ± 1.08	0.19	.85
Shell intactness rating	1.88 ± 2.10	0.89	.37
Shell entrance size	−1.20 ± 0.46	−2.63	**.0086**
Shrimp presence	1.74 ± 2.42	0.72	.47
Smooth versus rough shell	0.72 ± 1.16	0.62	.54
Dominant male (baseline) versus Female			
Intercept	4.08 ± 1.84	2.22	**.027**
Surface versus basement shell	−0.25 ± 0.85	−0.30	.77
Shell intactness rating	−2.62 ± 1.16	−2.25	**.025**
Shell entrance size	−1.63 ± 0.40	−4.08	**<.0001**
Shrimp presence	2.96 ± 1.75	1.69	.091
Smooth versus rough shell	0.90 ± 0.99	0.91	.36
Dominant male (baseline) versus Juvenile			
Intercept	6.13 ± 2.00	3.07	**.0022**
Surface versus basement shell	−0.95 ± 0.87	−1.09	.28
Shell intactness rating	−4.89 ± 1.25	−3.91	**<.0001**
Shell entrance size	−2.26 ± 0.43	−5.21	**<.0001**
Shrimp presence	1.94 ± 1.66	1.17	.24
Smooth versus rough shell	0.13 ± 1.10	0.12	.90
Subordinate Male (baseline) versus Female			
Intercept	4.44 ± 2.18	2.03	.042
Surface versus basement shell	−0.46 ± 0.86	−0.53	.60
Shell intactness rating	−4.50 ± 1.97	−2.28	**.023**
Shell entrance size	−0.43 ± 0.35	−1.20	.23
Shrimp presence	1.22 ± 2.18	0.56	.58
Smooth versus rough shell	0.18 ± 0.89	0.20	.84
Subordinate male (baseline) versus Juvenile			
Intercept	6.49 ± 2.30	2.83	**.0047**
Surface versus basement shell	−1.15 ± 0.87	−1.32	.19
Shell intactness rating	−6.77 ± 2.01	−3.37	**.0008**
Shell entrance size	−1.05 ± 0.38	−2.74	**.0061**
Shrimp presence	0.20 ± 2.29	0.09	.93
Smooth versus rough shell	−0.59 ± 1.02	−0.58	.56
Female (baseline) versus Juvenile			
Intercept	2.05 ± 1.28	1.61	.11
Surface versus basement shell	−0.69 ± 0.51	−1.35	.18
Shell intactness rating	−2.27 ± 0.64	−3.52	**.0004**
Shell entrance size	−0.63 ± 0.23	−2.69	**.0071**
Shrimp presence	−1.02 ± 1.32	−0.77	.44
Smooth versus rough shell	−0.77 ± 0.78	−0.98	.33

*Note*: Significant *p* values at α = .05 are in bold.

**FIGURE 5:**
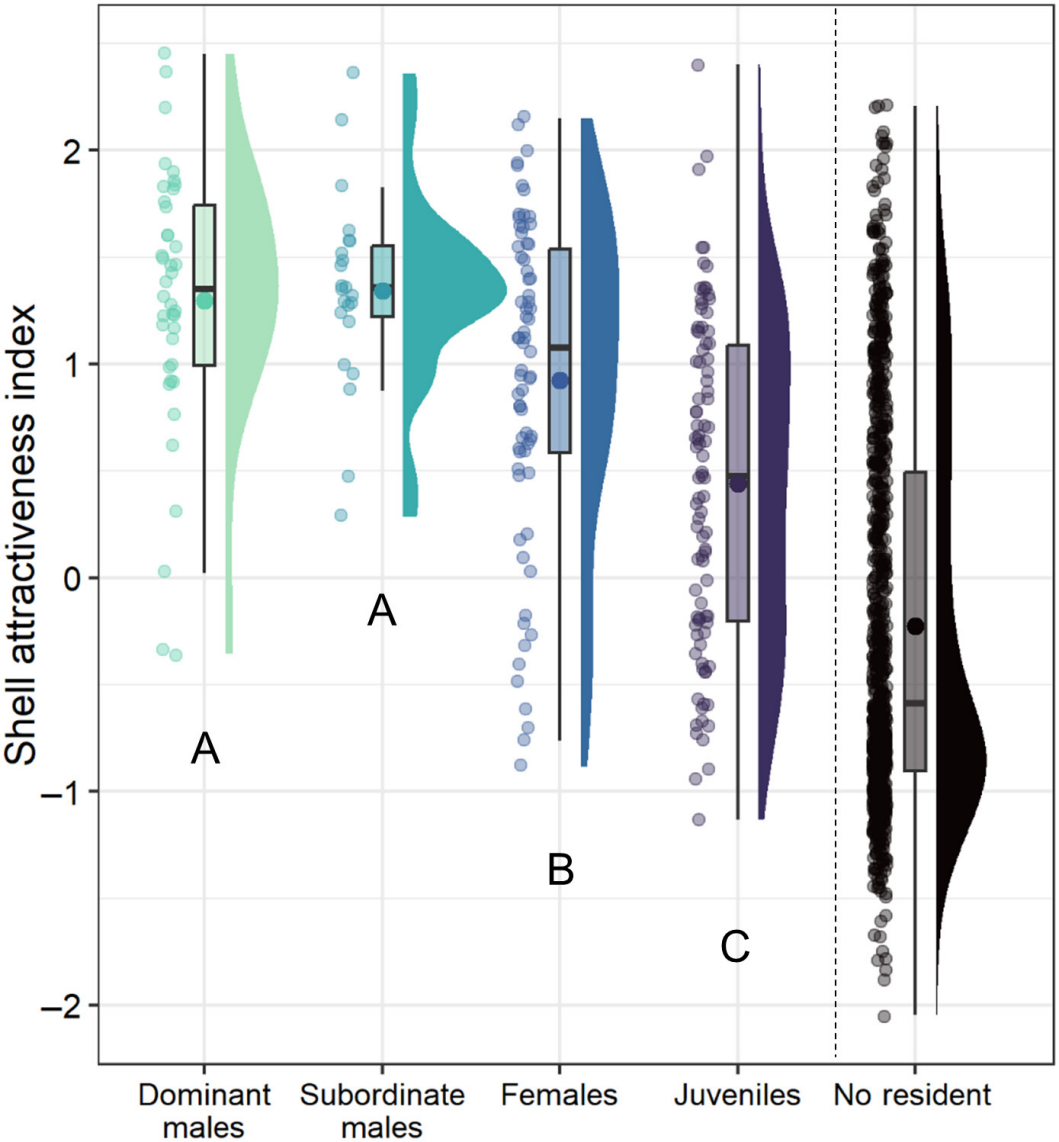
Shell attractiveness index (see Methods) compared across different types of occupants with a multinomial baseline‐category logit model. Unoccupied shells are also shown to the right of the vertical dashed line, but were not included in the multinomial model. Data are visualized as points on the left (jittered slightly to improve visibility), box plots in the middle, and density plots on the right. Boxplots show medians (horizontal bar), means (large dot), the first and third quartiles (box), and the range of data within 1.5 interquartile distances above and below the interquartile (whiskers). Significant differences between group member types are given by different capital letters below each distribution.

Our resource attractiveness index captured variation across shells in the probability that they would be occupied by a *N. multifasciatus* individual, relative to chance, given the distribution of resources that were available in each group's territory. Extreme positive values can therefore be interpreted as “the best of what is available,” while extreme negative values can be interpreted as “the worst of what is available.” As shell attractiveness increased, shells were more likely to be occupied by dominant or subordinate males rather than by females or juveniles, but shell attractiveness did not clearly separate where dominant males and subordinate males resided (Figure 5, Table 2).

**TABLE 2:** Output from a multinomial baseline‐category logit model, comparing *N. multifasciatus* dominant males, subordinate males, females, and juveniles in terms of their home shell's estimated attractiveness (see Methods).

Term	Estimate ± SE	*z*‐Value	*p*
Dominant male (baseline) versus subordinate male			
Intercept	0.062 ± 1.51	0.041	.97
Shell attractiveness index	0.48 ± 0.66	0.72	.47
Dominant male (baseline) versus female			
Intercept	1.91 ± 1.36	1.41	.16
Shell attractiveness index	−1.19 ± 0.44	−2.72	**.0065**
Dominant male (baseline) versus juvenile			
Intercept	2.46 ± 1.47	1.68	.093
Shell attractiveness index	−2.34 ± 0.46	−5.03	**<.0001**
Subordinate Male (baseline) versus female			
Intercept	1.85 ± 1.30	1.43	.15
Shell attractiveness index	−1.67 ± 0.60	−2.79	**.0052**
Subordinate male (baseline) versus juvenile			
Intercept	2.40 ± 1.41	1.70	.089
Shell attractiveness index	−2.82 ± 0.62	−4.55	**<.0001**
Female (baseline) versus juvenile			
Intercept	0.55 ± 1.08	0.51	.61
Shell attractiveness index	−1.15 ± 0.29	−4.01	**<.0001**

*Note*: Significant *p* values at α = .05 are in bold.

## 4 DISCUSSION

Animal decision‐making involves selecting one or more options out of numerous alternatives, which can be a challenging task if the alternatives differ with respect to many characteristics (Krieger et al., 2020). Individuals must therefore integrate information from multiple sources and make a judgment about the relative quality of their alternative options (Franks et al., 2003). As researchers, it can be challenging to pinpoint, a priori, which feature or features, approximate the quality of a resource. Though it is common practice for researchers to rely on their experience and intuition to choose which proxies to use, another way of tackling this problem, as we have done in this study, is to simultaneously assess how multiple resource features affect resource choice.

Our data highlight how multiple characteristics of a resource can jointly influence individuals' resource choices, underscoring the multivariate nature of resource quality. In the present field study, large, intact shells within a *N. multifasciatus* social group's territory were more likely to be occupied by group members than smaller, less intact shells. Despite shell size and intactness both having significant effects on shell occupancy, the 95% confidence intervals (CIs) for the parameter estimate of shell entrance size (scaled, 95% CI = 0.010–0.34) and shell intactness (scaled, 95% CI = 0.81–1.62) did not overlap suggesting that intactness was more important than size in determining occupancy (note that this difference stands for shells located at either the basement or surface levels). Previous studies on shell‐breeding Tanganyikan cichlids, for example, *L. callipterus*, have also uncovered preferences for large shells, but shell characteristics beyond size are rarely considered (Mitchell et al., 2014; Schütz & Taborsky, 2000, 2005). Both shelter size and degree of enclosure feature prominently in shelter choice decisions of other animals (e.g., hermit crabs, *Pagurus criniticornis*, Gorman et al., 2015; spiny lobsters, *Panulirus interruptus*, Spanier & Zimmer‐Faust, 1988; ants, *Leptothorax albipennis*, Franks et al., 2003; crayfish, *Orconectes rusticus*, Martin III & Moore, 2008; plainfin midshipman fish, *P. notatus*, Bose et al., 2018), suggesting that these characteristics are important for determining the quality of shelters across numerous taxa. However, it remains to be critically examined whether this focus on shelter size and intactness in resource value studies is due to the generalized importance of these characteristics or because they are noticeable, simple, and convenient proxy variables for researchers to manipulate in many study systems.

Our approach took a set of seven putatively important shelter features within the shell‐dwelling *N. multifasciatus* system and then demonstrated which ones had detectable effects on shelter occupancy. Overall, our data align with the laboratory results of Bose et al. (2020), where through the use of preference functions (see Rodríguez et al., 2013), the authors showed that both shell intactness and shell size were strongly preferred resource characteristics in *N. multifasciatus* (with intactness also being more important than size). In fact, larger and more intact shells were still preferred by large and small fish alike in Bose et al. (2020), though it is possible that the most preferred size of shell for adults differs from that of juveniles, which were not tested in that study. The authors of Bose et al. (2020) used intuition from years of experience working with their study system to choose which resource features to investigate. In our present study using field data and systematic testing of multiple shell features, we confirm that shell size and intactness are indeed important for shell choice in *N. multifasciatus*, and demonstrate the influence of additional shelter characteristics as well.

Whether a shell was located in the basement or surface level of a territory had a subtle influence on shelter choice in *N. multifasciatus*. Individuals were more forgiving of structural imperfections when shells were located in the basement of a territory rather than at the surface. This could be because basement shells were often surrounded by shell fragments and partially embedded in sand such that holes in their walls did not provide as much access, and hence vulnerability, from the outside. Alternatively, it may be more difficult for *N. multifasciatus* individuals to assess the intactness of shells that are located in the basement. Basement shells were also smaller on average than surface shells, were more likely to have smooth walls, more likely to house crabs, and had less sponge cover, emphasizing the complexity and multivariate nature of resource choice decisions in this system. Overall, juveniles were more likely to live in the basement relative to adult males, and nearly more likely than females. Juveniles of numerous species often reside in different microhabitats than their adult counterparts (e.g., *Diplodus* spp., Ventura et al., 2015; brown anole lizard, *Anolis sagrei*, Delaney & Warner, 2017), and can occupy different spatial positions within a social group as well (e.g., at the front edge or center of a group rather than at the rear or periphery, e.g., ring‐tailed coatis, *Nasua*, Hirsch, 2011; brown capuchin monkeys, *Cebus paella*, Janson, 1990). Given their differences, we suggest that surface shells and basement shells constitute distinct microhabitat options within *N. multifasciatus* territories, with basement shells potentially offering additional protection from predators since their entrances are less exposed to the water column and lake floor where predators hunt (e.g., *Mastacembelus* spp., *N. tetracanthus*, *Lepidiolamprologus cunningtoni*, *L. elongatus*).

Another influential resource characteristic uncovered by our study was the presence or absence of heterospecifics residing in (or on) the shells. Shell‐beds support a rich community of vertebrates and invertebrates, including shell‐dwelling or –breeding cichlids (Koblmüller et al., 2007; Sato & Gashagaza, 1997), juveniles of some non‐cichlid fishes that utilize the shells as shelters (personal observations), and an assemblage of sponges and crustaceans (see Cumberlidge & Von Sternberg, 1999; Erpenbeck et al., 2011; McGlue et al., 2010; Takahashi et al., 2012; Takahashi & Ota, 2016). We documented several instances of juvenile heterospecific fishes, as well as numerous instances of crabs (most likely juvenile *Platythelphusa armata*; Cumberlidge & Von Sternberg, 1999) and shrimp (most likely *Macrobrachium moorei* and/or *Limnocaridina spinipes*, Kamermans, 2019) residing inside of shells on *N. multifasciatus* territories. Crabs and shrimp have been previously reported in shells of a *Telmatochromis temporalis* dwarf morph located at a nearby shell bed in the south of Lake Tanganyika (Takahashi & Ota, 2016). We also found multiple instances of shells overgrown with sponges (see Erpenbeck et al., 2011). The frequency of interspecific shell sharing appears to differ greatly with species, but in general, the presence of heterospecifics had a negative effect on shell occupancy by *N. multifasciatus* (see model coefficients in Figure 3); *N. multifasciatus* never co‐resided with heterospecific fish (though we only observed *N* = 4 heterospecific fish within the shells), and rarely co‐resided with crabs or shrimp. Whether shell preferences of any of these heterospecifics conflict with those of *N. multifasciatus*, and the degree to which residency effects influence interspecific resource contests, would be interesting avenues for future research. In general, however, *N. multifasciatus* appear to have a limited ability to displace heterospecifics from a shell, which can render shells occupied by other species less useable. Juvenile crabs may also act as egg predators (Takahashi et al., 2012), which would strongly reduce the value of any shell they occupy for female fish seeking a brood chamber. The idea that shelter value can be influenced by the presence of heterospecifics has previously been investigated in the plainfin midshipman fish, *P. notatus* (Bose et al., 2018). In *P. notatus*, males build nests inside cavities beneath intertidal rocks. While males can expel many other intertidal organisms from their nests, they cannot remove all of them, particularly sessile species including sponges, tunicates, and bryozoans. These species occupy egg‐laying space that ultimately impairs the reproductive success of the resident fish, thereby reducing the overall quality of the nesting site (Bose et al., 2018).

In addition to demonstrating which shells within a *N. multifasciatus* territory were most likely to be occupied, our study also showed how the chosen shells were partitioned among different types of group member. As shell entrance sizes increased, the probability that a shell would be occupied by a dominant male rose significantly more than the probability that it would be occupied by subordinate males, females, or juveniles (Table 1). Similarly, the probability that a shell would be occupied by a dominant or subordinate male rose significantly more with increasing shell intactness relative to females and juveniles. Shell occupancy by juveniles was the least responsive to changes in shell entrance size and intactness relative to the other group member types. Such assortative resource use may reflect an ideal despotic distribution, generated by the most competitive group members (adult males) outcompeting less competitive group members (adult females followed by juveniles) from the highest quality resources. Thus, females and juveniles may be relegated to using “the best of what remains” after dominant and subordinate males have claimed their shells. Despotic distributions are common in nature and are characterized by dominant individuals forcing subordinate individuals to occupy inferior locations (Fretwell, 1972). For example, in several species of European vultures, adults are more likely to outcompete juveniles, displacing them from carrion resources (Moreno‐Opo et al., 2020). Furthermore, high‐ranking female Japanese macaques, *Macaca fuscata*, can force lower‐ranking females to use inferior food patches (Saito, 1996). Alternatively, such partitioning could occur if group members differed in their preferences (or preference strengths, see Rodríguez et al., 2013) for different shell characteristics. While our current study cannot definitively tease apart the roles of competitive ability and differentiated preferences on resource partitioning, previous laboratory trials showed that adult *N. multifasciatus* of both sexes similarly preferred larger and more intact shells, though preferences were weaker in smaller individuals (Bose et al., 2020).

When the probability that a resource will be chosen is influenced by multiple resource traits independently, it can be helpful to calculate one composite measure that captures the overall attractiveness of that resource. We therefore calculated an index to approximate the ultimate value or attractiveness of each shell in our dataset given its set of characteristics (e.g., shell entrance size, intactness, spatial position, etc.). We emphasize that this index is not intended to replace the widespread use of a few highly influential proxies for resource value. After all, measuring and comparing resources with respect to many features can be laborious and time‐consuming, and yield diminishing returns. But a composite index can be of general use as it would allow researchers to rank resources in order of their attractiveness (or “value”) even when those resources differ along various axes. Other dimension‐reducing statistical approaches do exist, for example, principal component analysis (PCA), in which multiple resource traits can be loaded together with a variable that describes how resources are used. However, in many animal systems, individuals can only choose from among the set of resources that they have immediate access to (i.e., from their local subset of resources rather than from the global set), which is a complexity that PCA does not accommodate but our approach does. In our resource attractiveness index, each resource characteristic exerts an influence on the final score proportional to its parameter estimate in the original model on which the index is based. As such, shell intactness is given a heavy weighting within our index, but the influence of other shell characteristics can also be observed (see Figure 5 in comparison to Figure 4a,b). Our attractiveness index utilized a binomial model to accommodate the binary state that each shell could be in (i.e., occupied vs. vacant). However, this index can also be extended to any type of starting model that suitably describes resource use in a given study system. For example, when resources are used discretely and repetitively, resource attractiveness scores could be calculated with starting models that are suitable for count data, where the conditional distribution of the response variable is given by a Poisson process. Similarly, resources that are used in a more continuous manner (e.g., time spent foraging on a resource patch) could be accommodated with a zero‐truncated Gaussian model. Ultimately, the choice of starting model will vary across study systems and researchers' individual expertise with a system will be integral for choosing which resource characteristics to include in the model. Finally, we used the choices of all individuals in each group to calculate our index, and we therefore produced attractiveness scores for each shell based on whether any *N. multifasciatus* individual was willing to occupy it (males, females, adults, juveniles, dominants, and subordinates alike). This index could in theory also be calculated for specific classes of individual separately, for example just for the resources used by dominant males. However, it should be advised that when doing so for less competitive individuals, for example, only for subordinates, the resulting index may no longer capture the most “valuable” or" “attractive” resources of the set, as subordinates are typically only able to choose the best of what remains when more dominant individuals have claimed the highest quality resources.

In this study, we equate the attractiveness of a resource to its quality. That is, we assume that resources are more likely to be chosen when they confer higher fitness benefits to the chooser. It is important to realize that this assumption is susceptible to evolutionary traps or sensory biases, which can sometimes cause individuals to be attracted to sub‐optimal resources. These are caveats that investigators will need to consider when choosing the set of characteristics to include in their resource attractiveness index calculations. The more closely this assumption holds, the more closely resource attractiveness scores should approximate resource quality.

We show that a social cichlid attends to multiple attributes of the shell resources on their territories, and that individuals of different sexes, age classes, and social statuses occupy shells with different combinations of characteristics (e.g., shell entrance size, intactness, and location in the territory). We also show how in some study systems, resource quality is not just a function of a resource's physical traits, but also of its spatial position in a landscape and of the community of heterospecifics that interact with it. Furthermore, we highlight how quantifying the attractiveness or the quality of a resource can benefit from a multivariate approach, and we give an example of a resource attractiveness index that incorporates information from any number of resource characteristics and can be flexibly adjusted to any study system. This index is not intended to replace the use of single proxy variables for resource quality (e.g., shelter size), but the utility of our index increases as single proxy variables become poorer approximators of resource quality. This should be of broad applicability for a wide variety of research questions involving resource choice or partitioning.

## AUTHOR CONTRIBUTIONS

**Aneesh P. H. Bose:** Conceptualization (lead); data curation (lead); formal analysis (lead); funding acquisition (supporting); investigation (lead); methodology (lead); project administration (equal); resources (equal); supervision (equal); visualization (lead); writing – original draft (lead); writing – review and editing (lead). **Tomas Brodin:** Funding acquisition (equal); resources (supporting); writing – review and editing (supporting). **Cyprian Katongo:** Project administration (equal); resources (equal); writing – review and editing (supporting). **Lwabanya Mabo:** Project administration (equal); resources (equal); writing – review and editing (supporting). **Alex Jordan:** Funding acquisition (equal); project administration (equal); resources (equal); writing – review and editing (supporting).

## CONFLICT OF INTEREST STATEMENT

The authors have no conflicts of interest to declare.

### OPEN RESEARCH BADGES

This article has earned Open Data badges. Data and materials are available at https://doi.org/10.17605/OSF.IO/2JMDB.

## Data Availability

All associated data and R code are available at Open Science Framework (DOI 10.17605/OSF.IO/2JMDB).
